# Pim1 promotes the maintenance of bone homeostasis by regulating osteoclast function

**DOI:** 10.1038/s12276-025-01421-4

**Published:** 2025-04-01

**Authors:** Jeongin Seo, Ryeojin Ko, Minhee Kim, Jeongmin Seo, Hana Lee, Doyong Kim, Woojin Jeong, Han Sung Kim, Soo Young Lee

**Affiliations:** 1 Department of Life Science, Ewha Womans University, Seoul, South Korea; 2 Multitasking Macrophage Research Center, Ewha Womans University, Seoul, South Korea; 3 Brain Korea 21 FOUR Program, LIFE Talent Development for Future Response, Ewha Womans University, Seoul, South Korea; 4 Department of Biomedical Engineering, Yonsei University, Wonju, South Korea

**Keywords:** Bone, Osteoporosis

## Abstract

The Pim1 (proviral integration site for Moloney leukemia virus 1) protein is a serine/threonine kinase that is essential for cell proliferation, apoptosis and innate immune responses. Here we show that Pim1 promotes osteoclast resorptive function without affecting osteoclast numbers. Specifically, we found that mice lacking Pim1 (*Pim1*^−/−^) developed increased trabecular bone mass and indices such as trabecular bone-mass density. This was due to the direct phosphorylation of TRAF6 by Pim1 in mature osteoclasts, which activated the Akt–GSK3β signaling pathway. This, in turn, promoted the acetylation and consequent stabilization of microtubules, which permitted the formation of the osteoclast sealing zone. In vivo experiments then showed that, when mice with lipopolysaccharide-induced bone loss or tumor-induced osteolysis were treated with SGI-1776, a Pim1 inhibitor that is more selective for Pim1, the bone loss was significantly ameliorated. Thus, Pim1 plays an important role in osteoclast function and may be a therapeutic target for bone-related diseases.

## Pim1 protein enhances osteoclast function and bone health

This study explores how a protein called Pim1 affects bone health. Bones constantly renew themselves through a process involving cells called osteoclasts, which break down old bone, and osteoblasts, which build new bone. When this balance is disrupted, it can lead to diseases such as osteoporosis. Researchers found that Pim1, a type of enzyme, plays a role in this process by affecting the stability of structures within osteoclasts called microtubules. The study used mice that lacked Pim1 and found they had stronger bones due to reduced bone breakdown. This was linked to changes in microtubule stability, influenced by Pim1 through a pathway involving other proteins, including Akt and TRAF6. The researchers also tested a drug called SGI-1776, which inhibits Pim1, and found that it could protect against bone loss in conditions such as inflammation and cancer.

This summary was initially drafted using artificial intelligence, then revised and fact-checked by the author.

## Introduction

To maintain a healthy skeleton, bone must continuously undergo remodeling. This primarily involves the resorption of old bone by osteoclasts and the creation of new bone by osteoblasts^1,2^. Any imbalance in this process can lead to substantial structural and functional abnormalities in the skeleton that can induce morbidity and reduce lifespan. Most skeletal disorders in adults, including osteoporosis, periodontal disease, rheumatoid and psoriatic arthritis, Paget’s disease and multiple myeloma, are caused by excessive resorption activity by osteoclasts^3^. This is also the case for tumor-associated osteolysis; bone is a common metastatic site for breast, lung and prostate cancer cells, which release osteoclast-activating cytokines and cause the resulting mature osteoclasts to resorb the bone matrix. The osteoclasts, in turn, release growth factors that promote tumor growth, thus establishing a vicious cycle of tumor proliferation and bone resorption^4,5^. Treatments such as bisphosphonates (Zometa), which block osteoclast-resorptive activity, and antibodies that neutralize the osteoclastogenesis-inducing cytokine RANKL (denosumab) are used to treat these disorders. However, although these osteoclast inhibitors reduce bone degradation, bone pain and the frequency of pathological fractures, they offer only symptomatic relief and do not restore the damaged bone^6,7^. Moreover, both long-term bisphosphonate treatment and denosumab can suppress bone remodeling, which can lead to the ‘frozen bone’ phenomenon; this compromises bone quality and increases the risk of skeletal fragility and osteonecrosis of the jaw^8–10^. Therefore, the search for new targets and therapeutics for bone diseases is ongoing.

Osteoclasts are bone-resorbing multinucleated cells that originate from hematopoietic precursors in the bone marrow^11^. Once activated by RANKL, osteoclasts rearrange their cytoskeleton to generate dot-like structural units called podosomes, which consist of an F-actin-enriched core that is surrounded by a loose F-actin network called the podosome cloud. The podosomes pack together densely to form a ring-like superstructure at the basal membrane that adheres tightly to the skeletal bone surface, thus generating a sealing zone (also known as a podosome belt) into which the osteoclast can secrete bone-degrading acids and proteolytic enzymes^12,13^. Notably, the organization of the podosomes in the sealing zone is sustained by a complex network of microtubules^14,15^. Numerous studies have shown that microtubules play vital roles in bone resorption. In particular, it has been shown that formation of the sealing zone requires microtubule stabilization. A key mechanism that regulates microtubule stability in osteoclasts is the acetylation of tubulin; this promotes the binding of microtubule-associated proteins to the microtubule, which halts its dynamic instability and thereby stabilizes it^16–20^. Tubulin acetylation is regulated negatively by the Rho–mDia2–HDAC6 pathway^16^ and positively by the Akt–GSK3β axis^20^; these mechanisms thus respectively inhibit and promote both sealing-zone formation and bone resorption.

Pim1 (proviral integration site for Moloney leukemia virus 1) protein is a member of the Pim family (Pim1, Pim2 and Pim3) of highly conserved serine/threonine kinases^21–23^. These kinases are constitutively active, and their activity is mainly regulated at the transcriptional and translational levels. Pim1 plays critical roles in multiple cellular functions, including apoptosis, proliferation, the cell cycle, migration, cell survival, osteoclastogenesis and the innate immune system^24–30^. It is also a potent oncogene that is overexpressed in various hematopoietic malignancies and solid tumors, including prostate cancer. Indeed, Pim1 has been shown to regulate the migration, proliferation and survival of prostate cancer cells^29–33^.

Here, we show with Pim1-knockout mice that Pim1 promotes microtubule acetylation and thereby enhances osteoclast resorptive function. Specifically, we found that Pim1-knockout mice exhibit increased trabecular bone mass and that this reflected decreased bone resorption rather than decreased osteoclast formation or increased osteoblast formation. It also associated with microtubule acetylation through the Akt–GSK3β pathway. Moreover, we observed that the Pim inhibitor SGI-1776 prevented lipopolysaccharide (LPS)-induced bone loss and tumor-associated osteolysis, which suggests that Pim1 may be a potential therapeutic target for bone resorption-related diseases.

## Materials and methods

### Mice

*Pim1*^−/−^ mice were generated as reported previously^28^. All animal experimental procedures were approved by the Institutional Animal Care and Use Committee of Ewha Womans University (no. 24-010).

### Cells

Bone-marrow-derived macrophages (BMMs) derived from 6–8-week-old C57BL/6 male mice were prepared as described previously^34^. They were cultured in in alpha-minimum essential medium (Hyclone) supplemented with 10% fetal bovine serum (Hyclone) and 1% penicillin–streptomycin (Hyclone). The HEK 293T cell line was used for immunoprecipitation experiments. Plat-E packaging cells were used to generate retroviruses. The HEK 293T and Plat-E cells were cultured in Dulbecco’s modified Eagle medium (Hyclone) supplemented with 10% fetal bovine serum and 1% penicillin–streptomycin. RM1 cells are prostate-derived murine cells that are often used as a model of prostate cancer.

### Reagent and antibodies

Recombinant human macrophage-colony stimulating factor (M-CSF) and RANKL were purchased from R&D Systems. SGI-1776 (no. S2193) was obtained from Selleck Chemicals. LPS (no. L2885) was purchased from Sigma-Aldrich. The tartrate-resistant phosphatase (TRAP) staining kit was purchased from FUJIFILM Wako Pure Chemical Corporation. Antibodies specific for p-AKT (no. 9271, 1:1,000), Akt (no. 9272, 1:1,000), p-GSK3β (no. 9322, 1:1,000), GSK3β (no. 9315, 1:1,000) and TRAF6 (no. 8028, 1:1,000) were obtained from Cell Signaling Technology. The anti-Atp6v0d2 antibody was provided by Y. Choi (University of Pennsylvania). The Flag-specific antibody was purchased from Sigma (no. F3156, 1:5,000). Horseradish-peroxidase-conjugated secondary antibodies (1:10,000) were obtained from The Jackson Laboratory. Antibodies to phosphoserine (no. sc-81514, 1:1,000), HA (no. sc-7392, 1:1,000), Myc (no. sc-40, 1:1,000), NFATc1 (no. sc-17834), β-actin (no. sc-87778, 1:1,000) and GAPDH (no. sc-87724, 1:1,000) were obtained from Santa Cruz Biotechnology. Antibodies to acetylated tubulin (no. T6793 1:1,000) were purchased from Sigma-Aldrich. Antibodies to tubulin (no. ab6160, 1:10,000) were obtained from Abcam.

### Plasmids

The pMX-IRES-EGFP plasmids containing Flag-tagged murine Pim1 and the pIRES-hrGFP-2a plasmid containing HA-tagged murine Pim1 were a gift from Dr. N. S. Kim (Chonnam National University Medical School, Gwangju, Republic of Korea). The pFlag-CMV2 plasmids containing murine TRAF6 or mutant TRAF6 were previously described^35^. The pCDNA3.1 plasmid containing HA-tagged AKT1 was a gift from Dr. J. H. Song (Yonsei University, Seoul, Republic of Korea), and Myc-tagged AKT1 was generated through PCR and cloned into the pUSE vector.

### μCT and histology

The hindlimb was scanned via microcomputed tompography (μCT) (Skyscan1176, Bruker microCT) with the following parameters: aluminum 1-mm filter, a voxel size of 18 μm, voltage of 75 kV, a scanning current of 333 μA, a resolution of 18 μm, an exposure time of 260 ms and a rotation step of 0.7 deg. The acquired raw scanning data were then reconstructed into two-dimensional cross-sectional gray-scale image slices by using NRecon (Brucker micro-CT, ver.1.6.9.3). The reconstructed images were geometrically aligned by using DataViewer (Brucker micro-CT, ver.1.5.1.2), after which the structural parameters of the tibia and femur were analyzed by CT Analyzer (CT-AN ver.1.10.9.0, Brucker). The following trabecular bone variables were determined: bone-mineral density (BMD) (g/cm^2^), which reflects the amount of bone mineral in the bone tissue; bone-volume fraction (BV/TV, %), which is the proportion of trabecular bone in the region of interest; trabecular thickness (Tb.Th, mm), which is the thickness of the trabeculae in the trabecular bone; trabecular separation (Tb.Sp, mm), which is the diameter of the cavities between the trabeculae; trabecular number (Tb.N, mm-1); and trabecular-bone pattern factor (Tb.Pf, per mm), which relates to connectivity. The following cortical bone variables were determined: BMD (g/cm^2^); bone volume (mm^3^); cross-sectional thickness (mm), which is the cross-sectional thickness of the cortical bone; and mean polar moment of inertia (mm^4^), which relates to the ability to resist shearing.

### Bone histology and histomorphometry

The bones were fixed in 10% formaldehyde at 4 °C for 48 h, decalcified in 0.5 M EDTA (pH 7.4) for 14 days, embedded in paraffin, cut into 5-μm-thick sections and stained with hematoxylin and eosin. The osteoclastic variables number of osteoclasts per unit of bone surface (N.Oc/BS, n/mm) and osteoclast surface per unit of bone surface (Oc.S/BS, %) and the osteoblastic variable number of osteoblasts per unit of bone surface (N.OB/BS, n/mm) were analyzed by using ImageJ (National Institutes of Health). For TRAP staining, the sections were incubated with TRAP at room temperature for 40 min by using the TRAP-staining kit (FUJIFILM). The TRAP-positive multinucleated ≥200-µm-diameter cells were counted.

### Enzyme-linked immunosorbent assay

Serum from wild-type (WT) and *Pim1*^−/−^ male mice was collected, and the concentrations of CTX-1 or PINP were measured by using the RatLaps CTX-1 Enzyme Immunoassay (EIA) (AC-06F1; Immunodiagnostic Systems (IDS)) or rat/mouse PINP Enzyme Immunoassay (EIA) (AC-33F1; IDS) according to the manufacturer’s instructions.

### Osteoclast differentiation

BMMs were cultured with M-CSF (30 g/ml) and RANKL (100 ng/ml) in 48-well culture plates. Mature osteoclasts were fixed with 4% formaldehyde for 10 min and stained for the presence of TRAP by using the TRAP-staining kit (FUJIFILM) according to the manufacturer’s instructions.

### RNA extraction and quantitative PCR

Total RNA was extracted by using TRIzol (iNtRON Biotechnology) and then reverse-transcribed to cDNA by using PrimeScript RT Master Mix (Takara) according to the manufacturer’s instructions. Reverse transcription Quantitative PCR (RT-qPCR) was performed by using the SensiFAST SYBR LO-ROX kit (Bioline) on the Step-One-Plus Real-Time PCR instrument (Applied Biosystem). The following gene-specific primers were used: Nfatc1 sense, 5′-CCAGAAAATAACATGCGAGCC-3′, antisense, 5′-GTGGGATGTGAACTCGGAAG-3′; Atp6v0d2 sense, 5′-CAGAGATGGAAGCTGTCAACATTG-3′, antisense, 5′-TGCCAAATGAGTTCAGAGTG-3′; TRAP sense, 5′-CAGAGATGGAAGCTGTCAACA TTG-3′, antisense, 5′-TGCCAAATGAGTTCAGAGTG-3′, Pim1 sense, 5′-CGCGACATCAAGGACGAGAACA-3′, β-actin sense, 5′-CATTGCTGACAGGATGCAGAAGG-3′, antisense, 5′-TGCTGGAAGGTGGACAGTGAGG-3′.

### Retroviral transduction

To generate retroviruses, supernatants were collected from Plat-E packaging cells that were transfected with the empty pMX-IRES vector, pMX-IRES-Flag-Pim1, pMX-puro-TRAF6 (WT) and pMX-puro-TRAF6 (S48A) plasmid and then filtered through a 0.45-μm filter. For retroviral transduction, BMMs were infected for 5 h with the viral supernatant in the presence of polybrene (Sigma-Aldrich). After 24 h, the infected BMMs were cultured with M-CSF (30 ng/ml) and RANKL (100 ng/ml) to induce them to differentiate into osteoclasts.

### Bone-resorption assay

Pre-osteoclasts were treated with M-CSF (30 ng/ml) and RANKL (100 ng/ml) for 2 days and then plated onto dentin slices (Immunodiagnostic Systems) in 96-well plates in the presence of M-CSF and RANKL. The dentin sections were stained with hematoxylin, and the resorption pits were visualized with an Olympus CKX53 inverted microscope with ToupTek Cam. The surface area occupied by the pits was quantified by using Image Pro Plus software (Media Cybernetic). Alternatively, slices were stained with wheat germ agglutinin (WGA) and 20 μg/ml WGA-Alexa 488 conjugate (W11261, Invitrogen) was used for 30 min to measure the depth of the uptake pits. The depth of the uptake pits was detected by confocal microscopy (LSM 880 with Airyscan, Karl Zeiss) and measured using IMARIS (Bitplane).

### Immunofluorescence assay

Pre-osteoclasts were placed on dentin slices with M-CSF (30 ng/ml) and RANKL (100 ng/ml), left for 3 days to induce their maturation into osteoclasts, fixed with 4% paraformaldehyde for 10 min, permeabilized with 0.2% Triton X-100 for 5 min and then incubated with Alexa Fluor 488-phalloidin (Invitrogen) for 1 h to visualize the F-actin. The nuclei were stained with DAPI for 10 min. Alternatively, BMMs were plated on glass slides in 24-well plates, cultured for 4 days in the presence of M-CSF (30 ng/ml) and RANKL (100 ng/ml), fixed with 4% paraformaldehyde for 10 min, permeabilized with 0.2% Triton X-100 for 5 min and incubated with Alexa Fluor 594-phalloidin (Invitrogen) for 1 h to stain the F-actin and then DAPI to stain the nuclei. In other experiments, the glass-slide osteoclasts were incubated with anti-tubulin or anti-acetylated tubulin antibodies followed by their secondary antibodies (labeled with Alexa Fluor 594 and Alexa Fluor 488, respectively) as well as Alexa Fluor 647-phalloidin and DAPI. All cells were then subjected to confocal microscopy by using Zeiss LSM 880, and fluorescence intensity per cell was measured using IMARIS.

### Immunoprecipitation and immunoblot analysis

HEK 293T cells were transfected with the indicated plasmids for 36 h by using the PEI transfection reagent (Sigam-Aldrich). BMMs were stimulated with M-CSF and RANKL for 3 days. Thereafter, the cells were lysed with RIPA buffer (50 mM Tris–HCl (pH 8.0), 150 mM NaCl, 1% NP-40, 0.1% SDS and 0.5% sodium deoxycholate) containing phosphatase and protease inhibitors. The lysates were immunoprecipitated by incubation with the indicated primary antibodies overnight at 4 °C and then incubated with protein G Sepharose (Millipore) or A Sepharose (Millipore) at 4 °C for 1 h with gentle shaking. The immunoprecipitated proteins were washed with lysis buffer, boiled in 2× SDS sample buffer, separated on SDS–polyacrylamide gels and then electrophoretically transferred onto polyvinylidene difluoride membranes (Millipore). The membranes were blocked with 5% bovine serum albumin and incubated with the indicated primary antibodies and the appropriate HRP-conjugated secondary antibodies. The proteins were detected by using an enhanced chemiluminescence detection kit (Bio-Rad).

### LPS-induced bone destruction model

Phosphate-buffered saline (PBS) or LPS (12.5 mg/kg body weight) was injected into the calvariae of 5-week-old male mice twice at 48-h intervals. Starting one day after the first LPS injection, the mice were also injected intracranially with SGI-1776 (2.5 and 10 mg/kg) or vehicle every day for 5 days. The mice were euthanized 4 days after the second LPS or PBS injection, and the calvarial bones were collected, fixed with 10% formalin for 24 h, decalcified for 7 days in 0.5 M ethylenediaminetetraacetic acid, embedded in paraffin and cut into 5-μm-thick sections that were stained for TRAP.

### CCK-8 assay

RM1 cell viability was assessed using the Cell Counting Kit-8 (CCK-8) assay kit according to the instructions. The cells were incubated for 4 h at 37 °C after adding the CCK-8 solution (10 μl). Absorbance was measured at 450 nm using a microplate reader.

### Intratibial implantation of RM1 prostate cancer cells

Seven-week-old male mice were anesthetized, and their right tibia was injected with 2 × 10^3^ RM1 cells in 20 μl PBS. Starting 2 days after this injection, the mice were injected intraperitoneally with SGI-1776 (10 mg/kg and 30 mg/kg) or vehicle every 2 days. The tumor burden was measured 9 days after the RM1 injection by histological analysis of the bone with hematoxylin and eosin. μCT was conducted 12 days after RM1 injection.

### Statistical analysis

All data are presented as mean ± s.d. and represent at least three independent experiments. Groups were compared by using two-tailed Student’s *t*-test or one-way or two-way analysis of variance (ANOVA) with Tukey’s multiple comparisons test. GraphPad Prism 8.0 software was used for data analysis.

## Results

### *Pim1*^*−/−*^ male mice exhibit increased trabecular bone mass in the femur and tibia

**Fig. 1:**
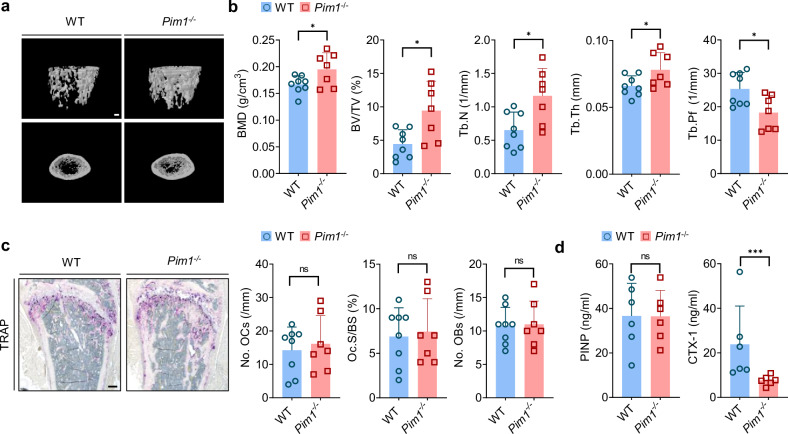
*Pim1*^−/−^ male mice exhibit increased bone mass in the femur. Eight-week-old WT and *Pim1*^−/−^ male mice were used. **a** Representative μCT images of the femurs. Scale bars, 500 μm. **b** Average values of the indicated μCT-determined bone indices. **c** Histomorphometry of the femurs after TRAP staining. Left: representative TRAP-stained femur images. Scale bars, 200 µm. Right: average histomorphometry-determined values of the indicated osteoclast and osteoblast variables. No.OCs, number of osteoclasts/bone perimeter; No.Obs, number of osteoblasts/bone perimeter. **d** Serum levels of PINP and CTX-1. In **a**–**c**, *n* = 7–8 per group. In **d**, *n* = 3 per group. The data are shown as mean ± s.d. Statistical analysis was conducted with two-tailed unpaired Student’s *t*-test. *P < 0.05, ***P < 0.001.

To determine the role of Pim1 in bone homeostasis in vivo, we used WT and *Pim1*^−/−^ male mice. *Pim1*^−/−^ mice have been reported previously to lack any distinct phenotypes^36^. Interestingly, at the age of 8 weeks, the *Pim1*^−/−^ mice showed increased femoral trabecular bone mass on μCT (Fig. 1a). Histology of the femur also showed that the *Pim1*^−/−^ mice had greater trabecular BMD, BV/TV, Tb.N and Tb.Th and decreased Tb.Pf (Fig. 1b). These findings were also observed for the tibial bone (Supplementary Fig. 1a, b). However, the cortical-bone phenotypes in the femur and tibia were not significantly altered by the lack of Pim1 (Supplementary Fig. 1c–f). Because bone growth is regulated by reciprocal and finely balanced interactions between osteoclasts, which degrade bone, and osteoblasts, which produce bone^37^, we next examined the osteoclast and osteoblast numbers in WT and *Pim1*^−/−^ mice. Significantly, femoral bone histology showed that the deletion of Pim1 did not alter the osteoclast and osteoblast numbers (Fig. 1c). Interestingly, however, while the *Pim1*^−/−^ mice had similar serum levels of the bone-formation marker PINP^38^, they had lower serum levels of the bone-resorption marker c-telopeptide of type I collagen (CTX-1)^38^ (Fig. 1d). Furthermore, we observed no significant differences in the bone phenotype between female WT and *Pim1*^−/−^ mice (Supplementary Fig. 2a–d). This result is probably due to the influence of female sex hormones, such as estrogen, which play a critical role in regulating bone homeostasis and osteoclast activity^39^. Thus, the elevated trabecular bone mass in *Pim1*^−/−^ male mice may be due to defective bone resorption by osteoclasts rather than altered osteoclast or osteoblast numbers.

### Pim1 regulates osteoclast function, not osteoclastogenesis or osteogenesis

**Fig. 2:**
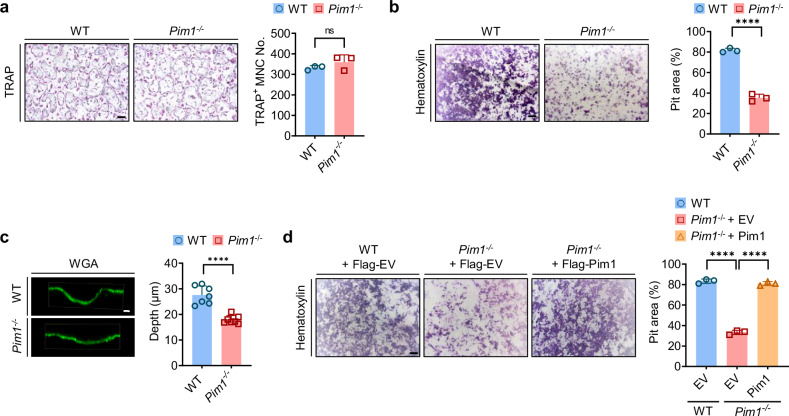
Pim1 regulates osteoclast function but not osteoclast formation. **a** WT and *Pim1*^−/−^ BMMs were cultured with M-CSF (30 ng/ml) and RANKL (100 ng/ml) in wells for 4 days. Left: representative image of the TRAP-stained WT and *Pim1*^−/−^ osteoclasts. Scale bars, 200 μm. Right: plot showing the counts of TRAP-positive multinucleated cells with ≥200-μm diameters. **b**, **c** WT and *Pim1*^−/−^ BMMs were cultured with M-CSF (30 ng/ml) and RANKL (100 ng/ml) on dentin slices for 5 days: hematoxylin staining was conducted to measure resorption-pit area (left: representative images of the hematoxylin-stained dentin (scale bar, 200 μm); right: plot showing the average resorption-pit area) (**b**); WGA staining to measure resorption-pit depth (left: representative images of the WGA-stained dentin (scale bar, 10 µm); right: plot showing the average resorption-pit depth) (**c**). **d** WT and *Pim1*^−/−^ BMMs were transfected with empty vector (EV) or Pim1 before being incubated with M-CSF (30 ng/ml) and RANKL (100 ng/ml) on dentin slices for 5 days. The dentin slices were then stained with hematoxylin. Left: representative image of the hematoxylin-stained dentin. Scale bar, 200 μm. Right: plot showing the average resorption-pit area. All data are representative of three independent experiments. The data are presented as mean ± s.d. Statistical analysis was conducted with two-tailed unpaired Student’s *t*-test and one-way ANOVA with Turkey’s multiple comparison test. ns, not significant. ****P < 0.0001.

It is already known that Pim1 is involved in osteoclastogenesis, as Kim et al. reported that the overexpression of Pim1 in pre-osteoclasts (BMMs) positively regulates RANKL-induced osteoclastogenesis in vitro^27^. To confirm the role of Pim1 in osteoclastogenesis, we cultured BMMs from WT and *Pim1*^−/−^ mice with M-CSF and RANKL in vitro to induce the BMMs to differentiate into mature osteoclasts. Surprisingly, the BMMs from the two mouse strains did not differ significantly in terms of the number of TRAP-positive multinucleated cells that arose (Fig. 2a). Moreover, the deletion of *Pim1* did not affect the mRNA or protein expression of Atp6v0d2 or NFATc1 by the M-CSF/RANKL-treated BMMs; these molecules play essential roles in the osteoclast fusion that leads to multinucleated mature osteoclasts^40^ (Supplementary Fig. 3a, b). By contrast, as expected, Pim1 expression was never observed in the *Pim1*^−/−^ BMMs (Supplementary Fig. 3a). We also observed that deleting *Pim1* did not modulate the M-CSF/RANKL-induced signaling pathway that leads to osteoclastogenesis (Supplementary Fig. 3c, d). These results suggest that Pim1 does not significantly affect osteoclast differentiation.

Bone growth can be induced by elevated osteoblast numbers, which can be driven by differentiation-inducing signals from osteoclasts^41^. To determine whether osteoblast numbers were elevated by *Pim1* deletion, calvaria osteoprogenitors from the WT and *Pim1*^−/−^ mice were induced to differentiate into osteoblasts. However, they demonstrated similar osteogenic differentiation, as shown by their alkaline phosphatase activity, the mineralization revealed by Alizarin Red S staining and their expression of osteoblast-specific genes (Supplementary Fig. 4a, b). Thus, the increased trabecular bone mass in the *Pim1*^−/−^ mice was not due to decreased osteoclastogenesis or increased osteoblastogenesis. Given that the *Pim1*^−/−^ mice had lower serum levels of CTX-1 but similar PINP levels (Fig. 1d), these data suggest that Pim1 plays a positive role in osteoclast degradation of trabecular bone.

To test this, we used the resorption-pit assay to examine osteoclast function in WT and *Pim1*^−/−^ mice. Thus, maturing osteoclasts were placed on dentin slices, after which the osteoclasts were removed and the slices were stained with hematoxylin to determine the resorption area. Indeed, the Pim1-deficient osteoclasts resorbed much less bone than the WT osteoclasts (Fig. 2b). This was also observed when the slices were incubated with WGA, which binds to the demineralized resorption lacunae left by osteoclasts^42^: WGA-fluorescein isothiocyanate staining showed that the Pim1-deficient osteoclasts left significantly shallower pits than the WT osteoclasts (Fig. 2c).

To confirm that Pim1 shapes osteoclast function, we overexpressed Pim1 in *Pim1*^−/−^ BMMs by retroviral transduction and treated them with M-CSF/RANKL. Indeed, the overexpression of *Pim1* in the Pim1-deficient osteoclasts caused them to recover their resorptive activity completely (Fig. 2d). Thus, Pim1 modulates bone homeostasis by regulating osteoclast activity rather than by shaping osteoclastogenesis or osteogenesis.

### Pim1 promotes normal actin cytoskeleton reorganization in osteoclasts by regulating microtubule acetylation

**Fig. 3:**
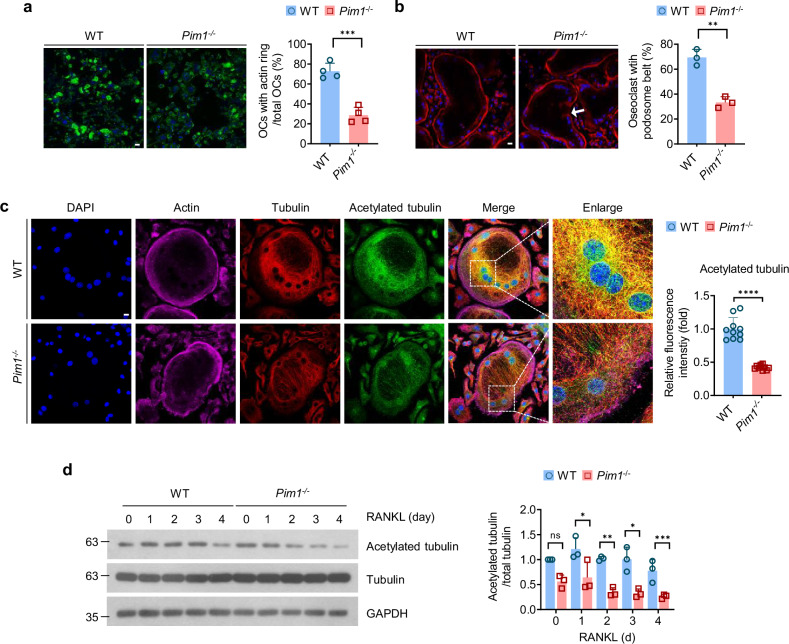
Pim1 promotes osteoclast cytoskeleton reorganization by inducing microtubule acetylation. **a**–**c**, WT and *Pim1*^−/−^ BMMs were cultured with M-CSF (30 ng/ml) and RANKL (100 ng/ml) for 4–6 days on dentin slices or glass slides: the cells on the dentin slices were stained with Alexa Fluor 488-phalloidin (left: representative images of the osteoclasts (scale bars, 200 μm); right: plot of the frequency of osteoclasts that bore an actin ring) (**a**); the cells on the glass slides were stained with Alexa Fluor 594-phalloidin (left: representative images of the osteoclasts (scale bars, 20 μm); right: plot of the frequency of osteoclasts that bore a podosome belt) (**b**); the cells on the glass slides were incubated with antibodies specific for tubulin or acetylated tubulin followed by Alexa Fluor 594 (bright red)- and Alexa Fluor 488 (green)-labeled secondary antibodies, respectively, and F-actin was also stained with Alexa Fluor 647-phalloidin (purple) (left: representative images (scale bars, 20 μm); right: mean intensity of acetylated tubulin per osteoclast (*n* = 10)) (**c**). **d** WT and *Pim1*^−/−^ BMMs were cultured with M-CSF (30 ng/ml) and RANKL (100 ng/ml) in wells for the indicated durations, and the total cell lysates were collected and immunoblotted with the indicated antibodies. Band intensity was determined with ImageJ. Left: representative immunoblot. Right: average amount of acetylated tubulin relative to total tubulin. All data are representative of three independent experiments. The data are presented as mean ± s.d. Statistical analysis was conducted with two-tailed unpaired Student’s *t*-test and two-way ANOVA with Tukey’s multiple comparison test. ns, not significant. *P < 0.05, **P < 0.01, ***P < 0.001.

Osteoclast resorption depends on the reorganization of the actin cytoskeleton, which clusters and then undergoes peripheral organization to form the F-actin ring that creates the sealing zone^43^. Because Pim1 has been found to regulate the actin cytoskeleton in prostate cancer cells, thus promoting their invasive motility^44^, and the *Pim1*^−/−^ BMMs were poorly resorptive (Fig. 2b, c), we hypothesized that Pim1 regulates osteoclast function by controlling the actin cytoskeleton reorganization. To test this, we generated WT and Pim1-deficient osteoclasts on dentin slices, permeabilized the cells and stained their F-actin with Alexa Fluor 488-phalloidin. Immunofluorescence analysis showed that the Pim1-deficient osteoclasts were less likely to bear F-actin rings (that is, sealing zones, also known as podosome belts) than the WT osteoclasts (Fig. 3a). This was also observed when we generated WT and Pim1-deficient osteoclasts on glass coverslips and stained them with Alexa Fluor 594-phalloidin: Pim1 deficiency greatly reduced the number of osteoclasts with a podosome belt (Fig. 3b).

The formation of the sealing zone is highly reliant on microtubules: as osteoclasts differentiate, microtubules extend toward isolated or clustered podosomes and eventually form a ring at the podosome belt^15^. This requires acetylation of the microtubules, which pauses their dynamic instability and stabilizes them^45^. We thus speculated that the poor ability of Pim1-deficient osteoclasts to form sealing zones was due to microtubule instability. To test this, Pim1-deficient and WT osteoclasts were matured on glass, permeabilized and incubated with antibodies against tubulin or acetylated tubulin. Immunofluorescence analysis revealed reduced microtubule acetylation and disruption of the microtubule architecture in the Pim1-deficient osteoclasts (Fig. 3c). Immunoblotting of these cells confirmed that Pim1 deficiency associated with lower tubulin acetylation levels at all time points during osteoclast differentiation (Fig. 3d). Thus, Pim1 appears to promote the actin-cytoskeleton reorganization needed to form the sealing zone by regulating microtubule acetylation.

### A Pim kinase inhibitor impairs cytoskeleton reorganization and downregulates osteoclast resorption

**Fig. 4:**
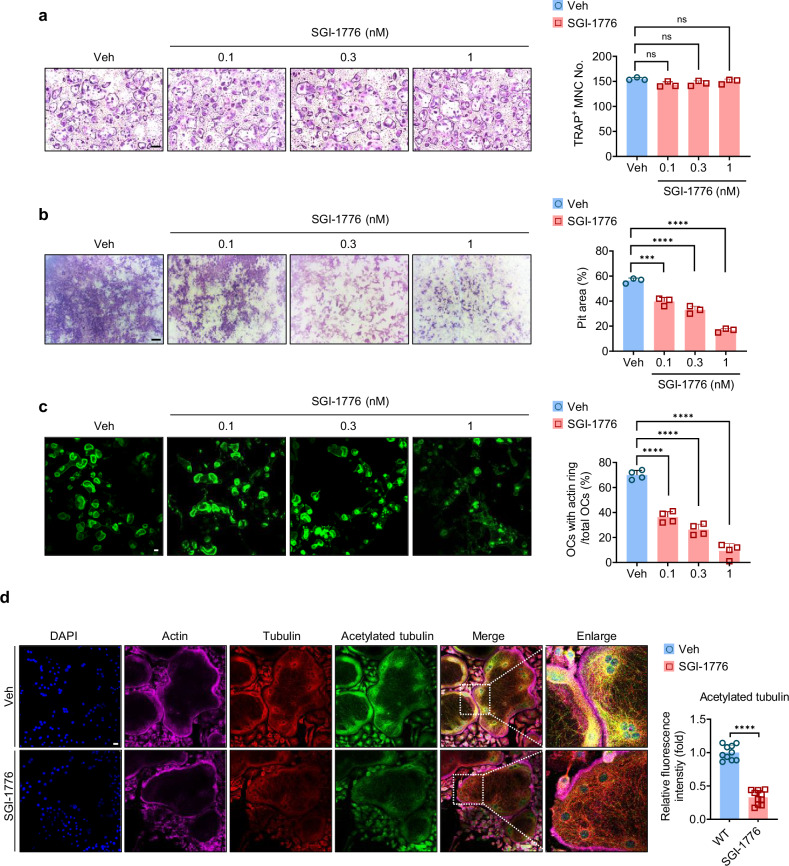
The SGI-1776 Pim1 inhibitor downregulates osteoclast resorption by blocking cytoskeleton reorganization. BMMs were incubated with SGI-1776 (0.1, 0.3 or 1 nM) for 4 days with M-CSF (30 ng/ml) and RANKL (100 ng/ml). **a** The cells were cultured as described in wells and then stained with TRAP. Left: representative images of the osteoclasts. Scale bars, 200 μm. Right: plot showing the average number of TRAP-positive multinucleated cells with ≥200-μm diameters. **b** The cells were cultured as described on dentin and then stained with hematoxylin. Left: representative images of the osteoclasts. Scale bars, 200 μm. Right: plot showing the average resorption-pit area. **c** The cells were cultured as described on dentin slices and stained with Alexa Fluor 488-phalloidin. Left: representative images of the osteoclasts. Scale bars, 200 μm. Right: plot showing the average frequency of osteoclasts with a normal actin ring. **d** The cells were cultured as described on glass slides, incubated with antibodies specific for tubulin and acetylated tubulin followed by Alexa Fluor 594 (bright red)- and Alexa Fluor 488 (green)-labeled secondary antibodies, respectively. The cells were also stained with Alexa Fluor 647-phalloidin (green) to indicate their F-actin. Left: representative images. Scale bars, 20 μm. Right: mean intensity of acetylated tubulin per osteoclast (*n* = 10) (right). All data are representative of three independent experiments. The data are presented as mean ± s.d. Statistical analysis was conducted with one-way ANOVA with Tukey’s multiple comparison test. ns, not significant. ***P < 0.001, ****P < 0.0001.

Pim1 is a serine/threonine kinase that phosphorylates various substrates, thereby regulating many cellular processes^22^. To determine whether the kinase activity of Pim1 is needed for its ability to regulate osteoclast function, BMMs were stimulated for 4 days with M-CSF and RANKL in the presence of SGI-1776; this is a Pim kinase inhibitor that preferentially inhibits Pim1 over Pim2 and Pim3 (the half maximal inhibitory concentration (IC_50_) values are 7, 363 and 69 nM, respectively)^46^. We first checked the impact of SGI-1776 on osteoclast formation and found it had no effect (Fig. 4a). By contrast, the inhibitor significantly decreased the the bone resorption-pit area in a dose-dependent manner (Fig. 4b). The inhibitor also reduced the number of osteoclasts with actin rings (Fig. 4c), decreased microtubule acetylation and disrupted the microtubule architecture (Fig. 4d). These findings are all similar to the effect of Pim1 deficiency. Thus, the kinase activity of Pim1 is needed for its role in regulating microtubule acetylation, thereby shaping osteoclast function.

### Pim1 promotes the Akt–GSK3β signaling pathway by phosphorylating TRAF6

**Fig. 5:**
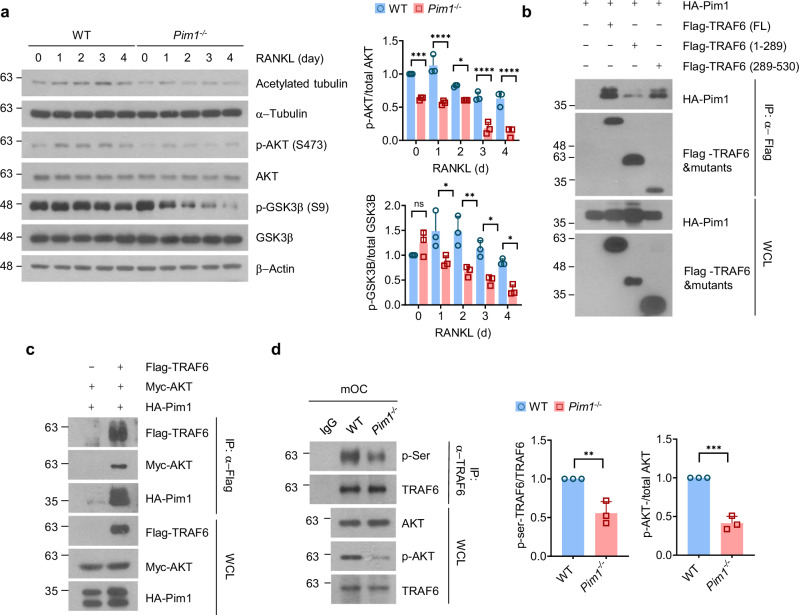
Pim1 promotes the Akt-signaling pathway by directly phosphorylating TRAF6. **a** WT and *Pim1*^−/−^ BMMs were cultured with M-CSF (30 ng/ml) and RANKL (100 ng/ml), and total cell lysates were collected at the indicated times and subjected to immunoblotting with the indicated antibodies. Band intensity was quantified with ImageJ. Left: representative immunoblot. Right: plots showing the mean phospho-Akt/total Akt ratio (top) and phospho-GSK3β/total GSK3β ratio (bottom). **b**, **c** HEK 293T cells were transfected with the indicated combinations of expression plasmids, followed by co-immunoprecipitation with an anti-Flag antibody. The proteins in the immunoprecipitates were detected by immunoblotting with the indicated antibodies. The expression levels of the transfected plasmids were verified by immunoblot analysis of the whole-cell lysates. Representative immunoblots are shown. **d** Cell lysates from mature WT and Pim1^−/−^ osteoclasts were subjected to immunoprecipitation with an anti-TRAF6 antibody. The proteins in the total cell lysates were detected by immunoblotting with the indicated antibodies. Band intensity was quantified with ImageJ. Left: a representative immunoblot. Middle: plot showing the average phospho-Akt/total AKT ratios. Right: plot showing the average serine-phosphorylated TRAF6 levels relative to total TRAF6 levels. All data are representative of three independent experiments. The data are presented as mean ± s.d. Statistical analysis was conducted with two-tailed unpaired Student’s *t*-test and two-way ANOVA with Tukey’s multiple comparison test. ns, not significant. *P < 0.05, **P < 0.01, ***P < 0.001, ****P < 0.0001.

It is well established that the Akt–GSK3β signaling axis positively regulates microtubule acetylation and stabilization, thereby promoting the bone-resorbing activity of osteoclasts. This axis involves the phosphorylation of Akt, which then phosphorylates the glycogen synthase kinase GSK3β. This prevents GSK3β from phosphorylating microtubule-associated proteins such as adenomatous polyposis coli: when adenomatous polyposis coli is phosphorylated by GSK3β, it displays poor binding to microtubules, which reduces microtubule stability, as shown by their decreased acetylation^20^. Notably, several studies have shown that, when Pim1 is blocked by a specific antibody or siRNA in various cancer-cell lines, the phosphorylation of Akt decreases^47,48^. Given that the kinase activity of Pim1 is needed for osteoclast function (Fig. 4), we hypothesized that Pim1 may regulate microtubule stability in osteoclasts by inducing Akt phosphorylation. To test this, we cultured BMMs from WT and *Pim1*^−/−^ mice with RANKL stimulation for the indicated times and conducted immunoblotting. Indeed, Pim1 deficiency significantly reduced Akt phosphorylation levels. The phosphorylation of GSK3β, which is the target of Akt, was also decreased, as were the acetylated-tubulin levels (Fig. 5a). Thus, Pim1 may stabilize microtubules and promote osteoclast function by inducing Akt phosphorylation.

It was unclear whether the phosphorylation event was directly mediated by Pim1 or involved other molecules. To determine whether Pim1 directly phosphorylates Akt, we overexpressed Flag-tagged Pim1 and HA-tagged Akt and performed immunoprecipitation and immunoblotting assays. However, our results showed that Pim1 did not interact with Akt (Supplementary Fig. 5a). With regard to the latter mechanism, a possible target of Pim1 is the E3 ligase TRAF6: it ubiquitinates Akt in various cells, including prostate cancer cells, when they are stimulated with insulin like factor-1 (IGF-1R). This ubiquitination event causes Akt to localize at the membrane and become phosphorylated^49^. Moreover, it is known that phosphorylation of TRAF6 can regulate downstream signaling pathways by enhancing or inhibiting its ubiquitination activity^50–52^. To test whether Pim1 phosphorylates TRAF6, which in turn promotes the phosphorylation of Akt activation, we overexpressed HA-tagged Pim1 with Flag-tagged TRAF6 and/or Myc-tagged Akt in HEK 293T cells and conducted immunoprecipitation and immunoblotting analyses. These analyses first showed that Pim1 interacted directly with full-length TRAF6 (Fig. 5b). To determine which TRAF6 domain is important for its interaction with Pim1, we constructed plasmids that expressed either the N-terminal ring and zinc-finger domains of TRAF6 amino acids 1–289) or the coiled-coil TRAF-N domain and the preserved TRAF-C domain (amino acids 289–530). Pim1 was found to interact with the latter but not the former, indicating that TRFA6 binds to Pim1 via its TRAF-N and/or TRAF-C domains (Fig. 5b). Next, we showed that TRAF6 forms a ternary complex with Akt and Pim1 (Fig. 5c). To determine whether Pim1 directly phosphorylates TRAF6 and thereby activates Akt, we assessed the effect of Pim1 deficiency on TRAF6 phosphorylation in mature osteoclasts. Indeed, the WT osteoclasts, but not the Pim1-deficient osteoclasts, demonstrated phosphorylation at the serine residue of TRAF6 (Fig. 5d). Moreover, the phosphorylation of Akt in the whole-cell lysate was decreased in the Pim1-deficient osteoclasts. However, we confirmed that the interaction between TRAF6 and AKT in Pim1-deficient osteoclasts does not show a significant difference. This observation suggests that Pim1 regulates AKT activation not by altering the physical interaction between TRAF6 and AKT but rather by modulating TRAF6 phosphorylation and, consequently, its functional activity (Supplementary Fig 5b). To identify the phosphorylation sites of TRAF6 mediated by Pim1, we constructed TRAF6 mutants. Among these, the mutation of serine 48 to alanine (TRAF6 S48A) significantly suppressed phosphorylation by Pim1 (Supplementary Fig. 6a). To investigate whether the serine 48 residue of TRAF6 is critical for microtubule acetylation, we overexpressed TRAF6 (WT) and TRAF6 (S48A) in BMMs via retroviral transduction, followed by treatment with M-CSF and RANKL. Notably, overexpression of TRAF6 (WT) enhanced microtubule acetylation in osteoclasts, whereas microtubule acetylation was impaired in osteoclasts overexpressing TRAF6 (S48A) (Supplementary Fig. 6b, c). Thus, Pim1 appears to control Akt signaling by directly phosphorylating TRAF6 at the serine 48 residue.

### SGI-1776 protects mice from inflammatory-bone destruction

**Fig. 6:**
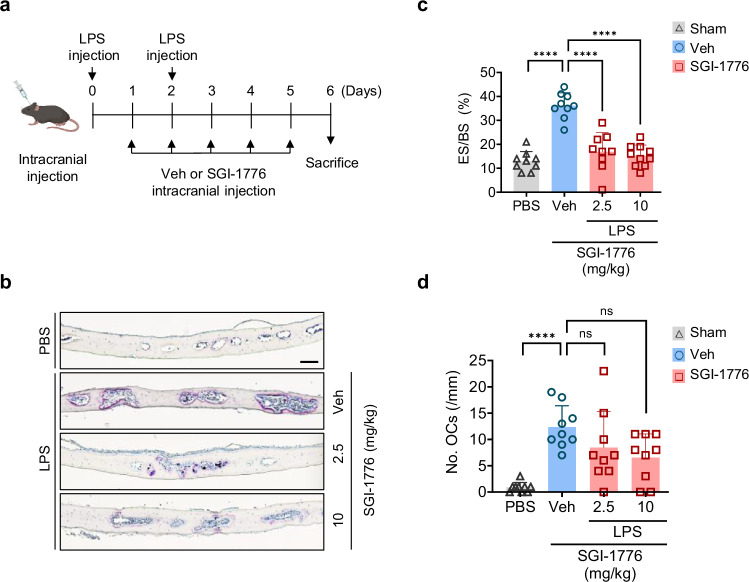
SGI-1776 inhibits LPS-induced bone resorption in vivo. **a** Schematic depiction of the experiment. Murine calvariae were injected twice with PBS (*n* = 9), LPS alone (12.5 mg/kg body weight alone (*n* = 9)), or LPS combined with either 2.5 mg/kg (*n* = 9) or 10 mg/kg body weight (*n* = 10) SGI-1776. **b**, **c** After euthanasia, the calvariae were sectioned and subjected to TRAP staining and histomorphometry: representative images of the TRAP-stained calvariae (scale bars, 100 μm) (**b**); the bone erosion was quantified and expressed as the percentage of eroded surface relative to total bone surface (ES/BS) (**c**). **d** Histomorphometry to determine the No.OCs. The results are presented as mean ± s.d. Statistical differences were analyzed by using one-way ANOVA with Tukey’s multiple comparisons test. ns, not significant. ****P < 0.0001.

To determine whether targeting Pim1 can reduce the excessive osteoclast activity that drives various bone diseases, we treated a murine model of osteoporosis-like disease with the Pim1 kinase inhibitor SGI-1776. The model involves calvarial bone injection with LPS, which induces inflammatory-bone destruction. Thus, the calvariae of 5-week-old male mice were injected twice with PBS or LPS at 48 h intervals. Starting 1 day after the first LPS injection, the calvariae were also injected daily with SGI-1776 at 2.5 or 10 mg/kg or vehicle for 5 days (Fig. 6a). Indeed, SGI-1776 treatment greatly reduced the bone erosion area. A mild dose effect was also observed (Fig. 6b, c). As expected, the LPS injection significantly increased the number of osteoclasts in the calvarial bone as shown in TRAP staining. However, SGI-1776 treatment did not significantly alter the osteoclast numbers (Fig. 6d). Thus, SGI-1776 protected the mice from inflammatory-bone destruction by dampening the excessive osteoclast activity rather than by inhibiting osteoclast formation.

### SGI-1776 blocks tumor-associated osteolysis by reducing both prostate cancer-cell survival and osteoclastic activity

**Fig. 7:**
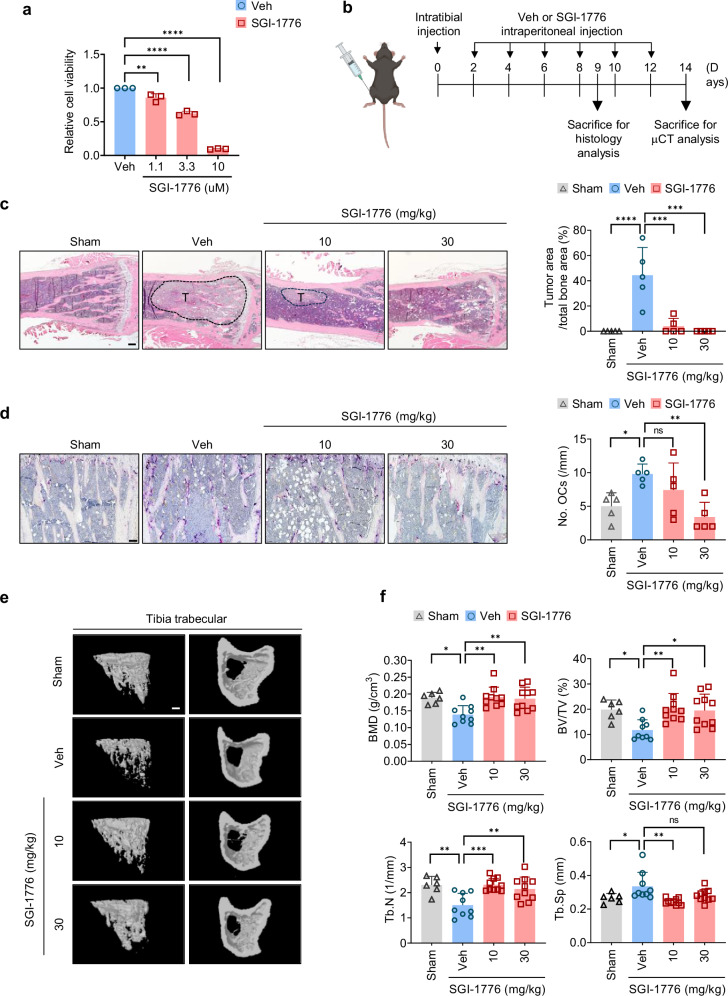
SGI-1776 inhibits prostate cancer growth and osteolytic activity in vivo. **a** The CCK-8 assay was used to test the relative viability of RM1 cells after 24 h treatment with SGI-1776 (1.1, 3.3 or 10 μM). **b** Schematic representation of the experiment. RM1 cells was injected into the tibiae of 7-week-old male mice. Starting 2 days later, the tibiae were injected with PBS, vehicle or either 10 mg/kg or 30 mg/kg body weight SGI-1776. **c**, **d** The tibiae were collected on day 9, sectioned and subjected to hematoxylin and eosin staining (**c**) or TRAP staining (**d**) with histomorphometry to determine the tumor area relative to total bone area and the No.OCs) (sham, *n* = 5; vehicle, *n* = 5; 10 mg/kg body weight SGI-1776, *n* = 5, 30 mg/kg body weight SGI-1776, *n* = 5). **e**, **f** The tibiae were collected on day 14 and subjected to µCT: representative µCT images (scale bars, 500 μm) (**e**); quantitative µCT analysis of tibial trabecular bone variables (sham, *n* = 6; vehicle, *n* = 9; 10 mg/kg body weight SGI-1776, *n* = 10; 30 mg/kg body weight SGI-1776, *n* = 10) (**f**). The results are presented as mean ± s.d. Statistical differences were analyzed by using one-way ANOVA with Tukey’s multiple comparisons test. ns, not significant. *P < 0.05, **P < 0.01, ***P < 0.001, ****P < 0.0001.

Previous studies have reported that Pim1 facilitates the progression of prostate cancer by promoting the migration, proliferation and survival of the cancer cells. It is therefore a promising anti-cancer treatment target^31–33,53^. Prostate cancer commonly metastasizes to bone and causes tumor-associated osteolysis^4,5^. Because we observed that inhibiting Pim1 diminishes osteoclast activity, we asked whether treating a mouse model of prostate cancer cell-associated osteolysis with SGI-1776 can reduce both the prostate cancer-cell proliferation and the osteolytic activity of osteoclasts. Thus, we established a model of prostate cancer-associated osteolysis by injecting the tibias of 7-week-old male C57BL/6 mice with the prostate cancer-cell line RM1, which is the cell line from C57BL/6 mice. When RM1 cells were treated with SGI-1776 in vitro, their proliferation dropped significantly in a dose-dependent manner as shown by using the CCK-8 assay (Fig. 7a). Thus, SGI-1776 can block RM1 proliferation. To assess the effect of SGI-1776 in vivo, the mice were injected intraperitoneally with 10 or 30 mg/kg inhibitor or vehicle every 2 days, starting 2 days after the RM1 injection. The mice were euthanized on day 9 for histology of the tibial bone and on day 14 for μCT (Fig. 7b). The histology showed that SGI-1776 greatly reduced the tumor area (Fig. 7c). The 10 mg/kg concentration of SGI-1776 did not significantly affect osteoclast numbers, but the 30 mg/kg dose did (Fig. 7d). The μCT analysis showed destruction of the tibial bone in the vehicle group and strong amelioration of this effect in the SGI-1776-treatment groups (Fig. 7e). Analysis of the bone architecture indicated that the SGI-1776 treatment protected the mice from cancer cell-associated loss of trabecular BMD, BV/TV and Tb.N. The treatment also reduced the Tb.Sp and Tb.Pf (Fig. 7f). However, the cortical-bone phenotypes in the femur and tibia were not significantly altered by the SGI-1776 treatment (Supplementary Fig. 7a, b). Thus, the SGI-1776 treatment greatly protected the bone architecture of the mice from tumor-associated osteolysis. To evaluate differences in the extent of prostate cancer-induced osteolysis between WT and *Pim1*^−/−^ mice, we injected RM1 cells into both groups. The histology revealed that tumor area was reduced in *Pim1*^−/−^ mice compared with WT mice, although the reduction was less than that observed with SGI-1776 treatment (Supplementary Fig. 8a). However, there was no difference in osteoclast number between WT and *Pim1*^−/−^ mice (Supplementary Fig. 8b). Taken together, these findings suggest that SGI-1776 can synergistically treat prostate cancer cell-induced osteolysis by reducing both the survival of the prostate cancer cells and their ability to stimulate osteoclastic activity.

## Discussion

The present study suggests that Pim1 is a regulator of osteoclast function. Specifically, we first observed that, at the age of 8 weeks, *Pim1*^−/−^ male mice had increased trabecular bone mass and indices such as trabecular BMD. We then found that Pim1 does not shape osteoclast or osteoblast formation; rather, it regulates osteoclast function by driving the cytoskeleton rearrangement that is needed to form the sealing zone. The underlying mechanism involves Pim1-mediated phosphorylation of TRAF6, which then induces downstream Akt phosphorylation. The activated Akt then stabilize the microtubules, as shown by increased microtubule acetylation. This microtubule stability permits the formation of the sealing zone and effective bone resorption. Significantly, our in vivo experiments indicated that SGI-1776, a Pim kinase inhibitor that is particularly effective with Pim1, could protect mice from progressive inflammatory-bone disease and tumor-induced osteolysis. Because Pim1 affects only the last step of osteoclast activity, rather than osteoclast or osteoblast formation, it may be a candidate drug target that could avoid the side effects of bisphosphonates and denosumab^6,7^.

Kim et al. found, using retroviral-gene transduction and small-interfering RNA systems, that Pim1 positively regulates RANKL-induced osteoclastogenesis^27^. However, the effects of Pim1 in vivo in osteoclast are still unknown. In this study, we were not able to replicate these findings: when BMMs from WT and *Pim1*^−/−^ mice were cultured with M-CSF and RANKL in vitro, they produced equivalent numbers of TRAP-positive multinucleated osteoclasts, and *Pim1* deletion did not affect the expression of Atp6v0d2, NFATc1 or M-CSF/RANKL-induced signaling pathway molecules.

Our in vitro data showed that Pim1 probably mediates its positive regulatory role in osteoclast resorption by controlling Akt activity. Specifically, Pim1 binds directly to TRAF6 in a ternary complex together with Akt: the binding of TRAF6 to Pim1 depends on the TRAF-N and TRAF-C domains and results in TRAF6 phosphorylation at serine residues. We showed that, once Pim1 binds to TRAF6, it phosphorylates it. This, in turn, induces Akt phosphorylation, thereby causing Akt to phosphorylate GSK3β, which is the major substrate of Akt and participates in many cellular processes^46^. We found that the Akt–GSK3β axis can be regulated by TRAF6, which is consistent with the literature on the role of Pim1 in cancer. Chauhan et al. showed that the improved survival is due in part to direct Pim1-mediated phosphorylation of GSK3β; this drives the accumulation of lipid droplets, which constitutes a survival advantage for the cancer cells during nutrient stress^31,54^. Several studies also show that Pim1 can indirectly regulate Akt phosphorylation in various cancers, and that this associates with improved survival and proliferation. Specifically, Sagar et al. reported that lower Pim levels associate with Akt inactivation as well as inhibition of the ubiquitin ligase MDM2, which then stabilize p53 and suppress cancer-cell apoptosis^48^. Moreover, Jiang et al. observed that the Pim inhibitor SMI-4a blocks the phosphorylation of Akt, as well as of PI3K and mTOR, in non-small cell lung cancer. This inhibition of the PI3K/AKT/mTOR pathway suppresses the proliferation of the cancer cells as well as elevating their apoptosis^55^. Similarly, Hu et al. showed that decreasing Pim-1 expression levels with a specific monoclonal antibody associated with reduced Akt phosphorylation at Ser473 in prostate cancer and increased cancer-cell apoptosis^47^.

The role of the E3 ligase TRAF6 in the ability of Pim1 to control Akt activity is also supported by the literature. Specifically, Yang et al. found that direct ubiquitination of Akt by TRAF6 is critical for the phosphorylation of Akt that is driven by growth-factor stimulation^49^. It should be noted that the phosphorylation of TRAF6 can either promote its ubiquitination activity or inhibit it. For example, when TRAF6 is phosphorylated by the germinal-center kinase MST4 at Thr463 and Thr486, it fails to oligomerize and ubiquitinate K63, which, in turn, blocks NF-κB signaling^51^. By contrast, phosphorylation of TRAF6 by the serine/threonine kinase RSK2 at Ser46, Ser47 and Ser48 promotes the K63 ubiquitination activity of TRAF6, which, in turn, drives LPS-induced inflammatory signaling^52^. Similarly, when IKKε phosphorylates TRAF6 at five serine residues (129, 188, 268, 279 and 324), it protects TRAF6 from basal ubiquitin/proteasome-mediated degradation, which, in turn, promotes TRAF6 signaling^50^. In our case, Pim1 also appears to promote TRAF6 activity and its ability to drive Akt phosphorylation by phosphorylating TRAF6 on serine residues.

This study examined the role of Pim1 in tumor-induced osteolysis by using SGI-1776. SGI-1776 is a small-molecule drug from the imidazo[1,2-b] pyridazine class. It is a potent ATP-competitive inhibitor of the Pim1, Pim2 and Pim3 kinases, but because its IC_50_ values are 7, 363 and 69 nM, respectively, it is particularly effective with Pim1 (ref. ^46^). Notably, our present study showed that SGI-1776 (2.5–10 mg/kg when injected in affected bone, and 10–30 mg/kg when injected intraperitoneally) effectively blocked osteoclast resorptive activity. Thus, it is possible that SGI-1776 could play a role in alleviating osteoclastic bone diseases.

In conclusion, our study provides several findings. First, Pim1 can regulate microtubule acetylation and stabilization, thereby driving the cytoskeletal rearrangement needed to generate the sealing zone and resorptive activity of osteoclasts. Second, the Akt–GSK3β-mediated acetylation of microtubules is driven by TRAF6 phosphorylation. Third, Pim1 can directly phosphorylate TRAF6, which appears to promote its ability to regulate the Akt–GSK3β axis. Fourth, the Pim inhibitor SGI-1776 effectively prevented inflammatory or tumor-induced osteolytic bone loss. Thus, Pim1 appears to play an important role in bone-mass regulation and may be a promising therapeutic target for bone-related diseases.

## Supplementary information

Supplementary Information
