# Supplementary material for: Pim1 promotes the maintenance of bone homeostasis by regulating osteoclast function
Source: Exp Mol Med. 2025 Apr 1;57(4):733–44. doi: 10.1038/s12276-025-01421-4 (PMC12046003; doi:10.1038/s12276-025-01421-4)
Supplement: Supplementary file 1 — Supplementary Information [file 12276_2025_1421_MOESM1_ESM.pdf]

Supplementary Information

Pim1 promotes the maintenance of bone homeostasis by regulating osteoclast function

Jeongin Seo[1,2,3], Ryeojin Ko[1,2], Minhee Kim[1,2,3], Jeongmin Seo[1,2], Hana Lee[4], Doyong Kim[4], Woojin Jeong[1,2,]3, Han Sung Kim[4] and Soo Young Lee[1,2,3*]

[1]Department of Life Science, Ewha Womans University, Seoul, 03760, South Korea

[2]Multitasking Macrophage Research Center, Ewha Womans University, Seoul, 03760, South Korea

[3]Brain Korea 21 FOUR Program, LIFE Talent Development for Future Response, Ewha Womans University, Seoul, 03760, South Korea

[4]Department of Biomedical Engineering, Yonsei University, Wonju, 26493, South Korea

*Correspondence should be addressed to:

S.Y. Lee, Multitasking Macrophage Research Center, Ewha Womans University, Seoul, 03760, South Korea. Tel: 82-2-3277-3770; Fax: 82-2-3277-3760; E-mail: leesy@ewha.ac.kr

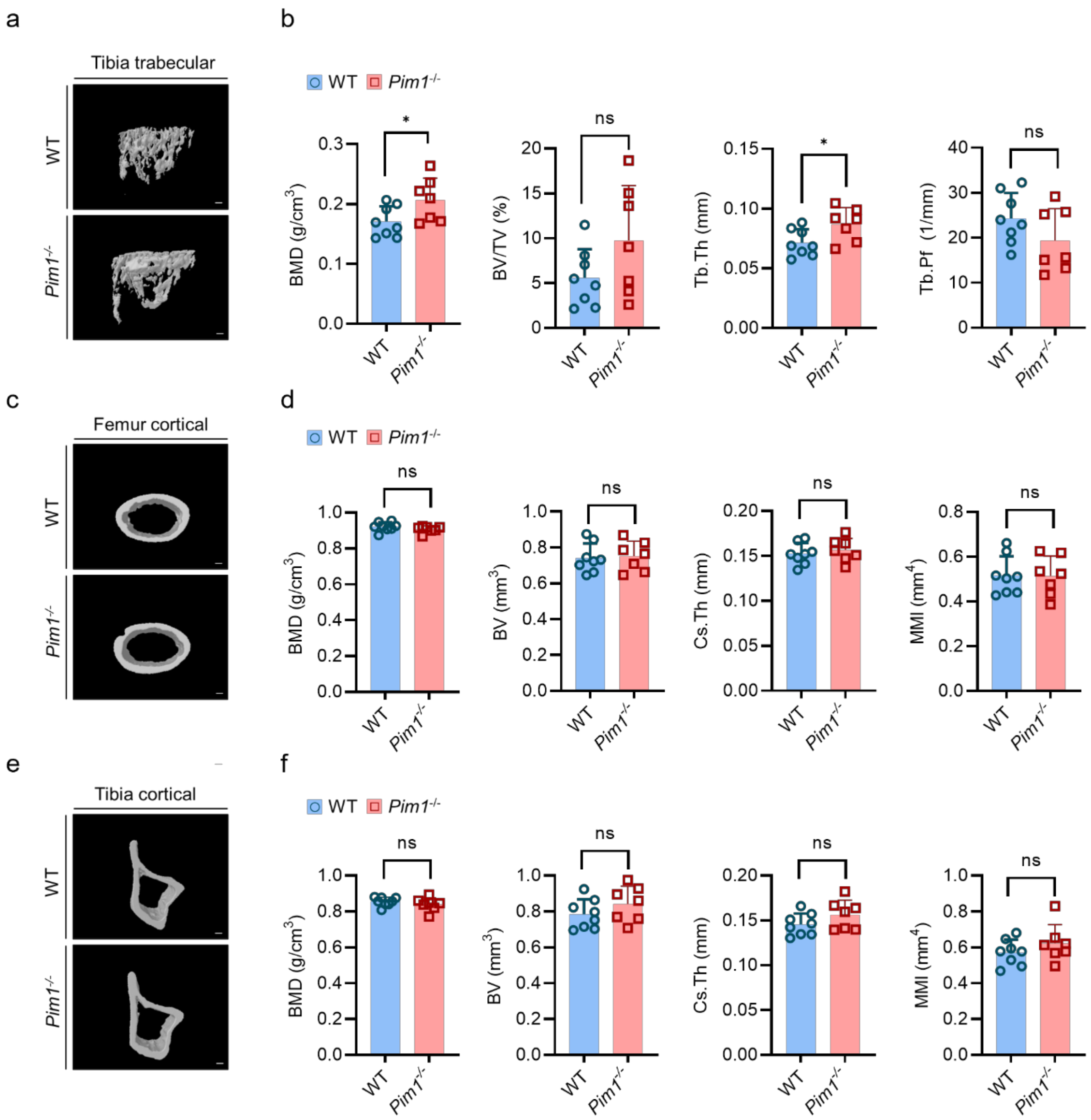

Supplementary Fig. 1. *Pim1*$^{-/-}$ mice show increased trabecular, but not cortical, bone mass in the tibiae.

Eight-week-old WT (n=8) and *Pim1*$^{-/-}$ (n=7) male mice were used. (**a, c, e**) Representative μCT images of the (**a**) trabecular bone in the tibiae, (**c**) cortical bone in the femur, and (**e**) cortical bone in the tibiae. Scale bars, 500 μm. (**b**) Quantitative μCT analysis of trabecular-bone variables in the tibiae. BMD=trabecular bone-mineral density; BV/TV=trabecular bone-volume density; Tb.Th=trabecular thickness; Tb.Pf=trabecular pattern factor. **(d, f)**

Quantitative μCT analysis of cortical-bone variables in the (**c**) femur and (**f**) tibiae. BMD=cortical bone-mineral density; BV=cortical bone volume; Cs.Th=crossectional cortical thickness; MMI=mean polar moment of inertia.The data are presented as mean ± SD. Statistical analysis was conducted with two-tailed unpaired Student's t-test. ns, not significant. *$p<0.05$.

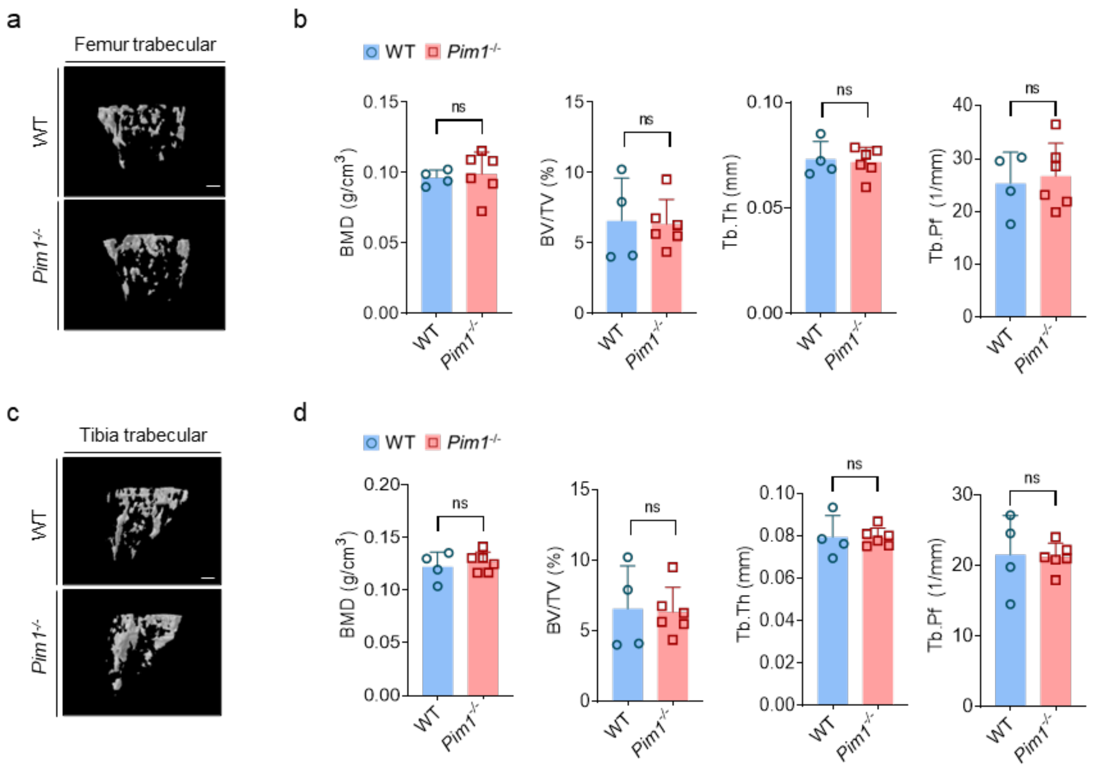

Supplementary Fig. 2. *Pim1*$^{-/-}$ female mice showed no difference in bone phenotype.

Eight-week-old WT (n=4) and *Pim1*$^{-/-}$ (n=6) female mice were used. (**a, c**) Representative μCT images of the (**a**) trabecular bone in the femur, (**c**) trabecular bone in the tibiae. Scale bars, 500 μm. (**b,d**) Quantitative μCT analysis of trabecular-bone variables in the femur and tibiae. BMD=trabecular bone-mineral density; BV/TV=trabecular bone-volume density; Tb.Th=trabecular thickness; Tb.Pf=trabecular pattern factor. The data are presented as mean ± SD. Statistical analysis was conducted with two-tailed unpaired Student's t-test. ns, not significant. *p<0.05.

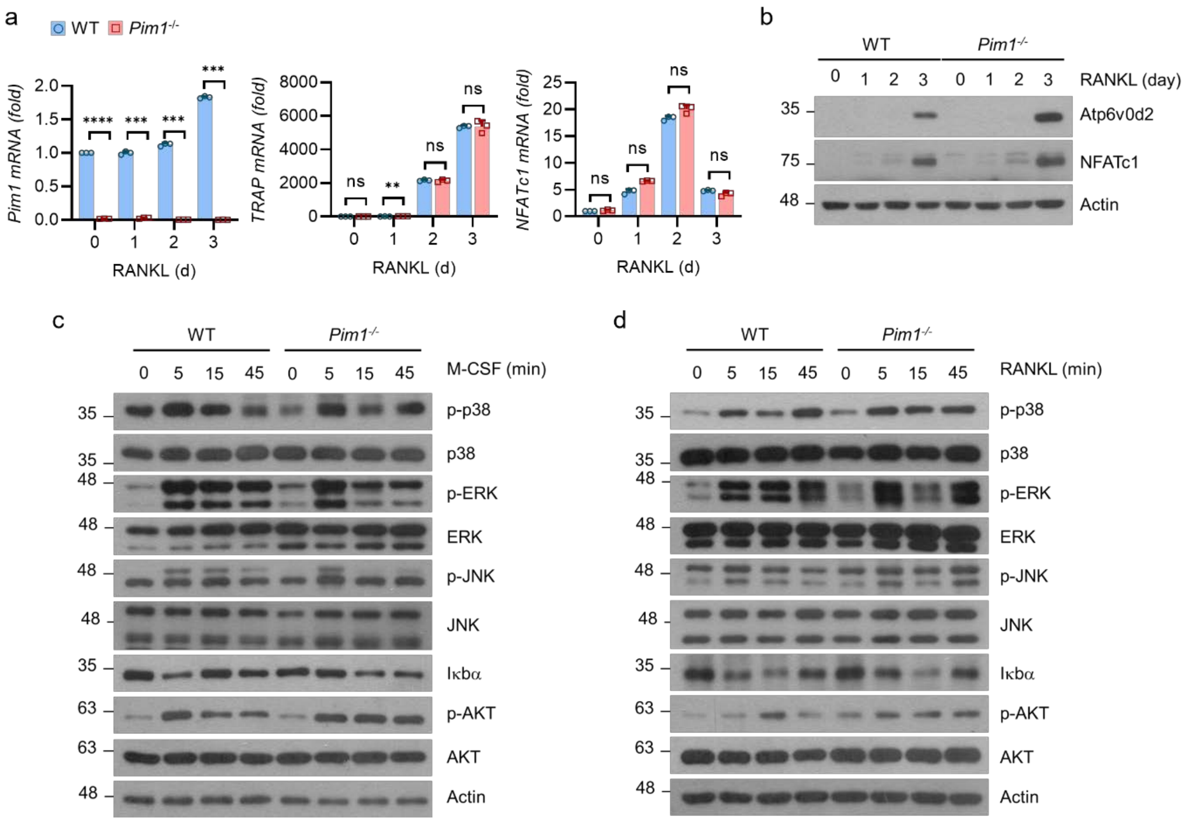

Supplementary Fig. 3. Pim1 does not affect osteoclast differentiation.

(**a, b**) WT and *Pim1*$^{-/-}$ BMMs were stimulated with M-CSF (30 ng/ml) and RANKL (100 ng/ml) for the indicated times, harvested, and subjected to **(b)** RT-qPCR analysis of the indicated molecules and **(b)** immunoblot analysis with the indicated antibodies. (**c, d**) BMMs derived from WT and *Pim1*$^{-/-}$ were starved for 6 hours and then stimulated with M-CSF (30 ng/ml) **(c)** and RANKL (500 ng/ml) **(d)** for the indicated times. The proteins were detected by immunoblotting with the indicated antibodies. The data are presented as mean ± SD. Statistical differences were analyzed by using one-way ANOVA with Tukey's multiple comparisons test. ns, not significant. **p<0.01; ***p<0.001; ****p<0.0001.

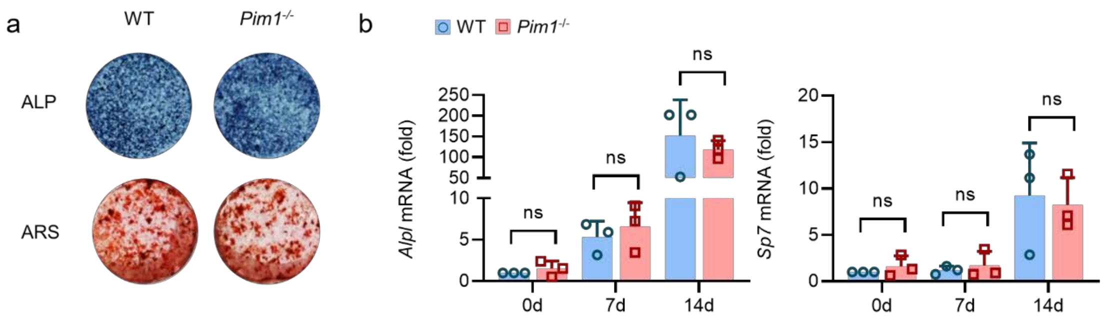

Supplementary Fig. 4. Pim1 does not affect osteoblast differentiation.

(**a, b**) Primary calvarial osteoblasts derived from WT and *Pim1*$^{-/-}$ were cultured with osteogenic medium (α-MEM containing 50 mg/ml ascorbic acid, 1 mM β-glycerophosphate, 10 nM dexamethasone and 50 ng/ml rhBMP-2) for 1 and 2 weeks and stained with ALP and Alizarin red (ARS). (**a**) Representative images of the osteoblasts in the wells. **(b)** mRNA expression of the indicated osteoblast marker genes, as determined by RT-qPCR. The data are presented as mean ± SD. Statistical analysis was conducted with two-tailed unpaired Student's t-test. ns, not significant.

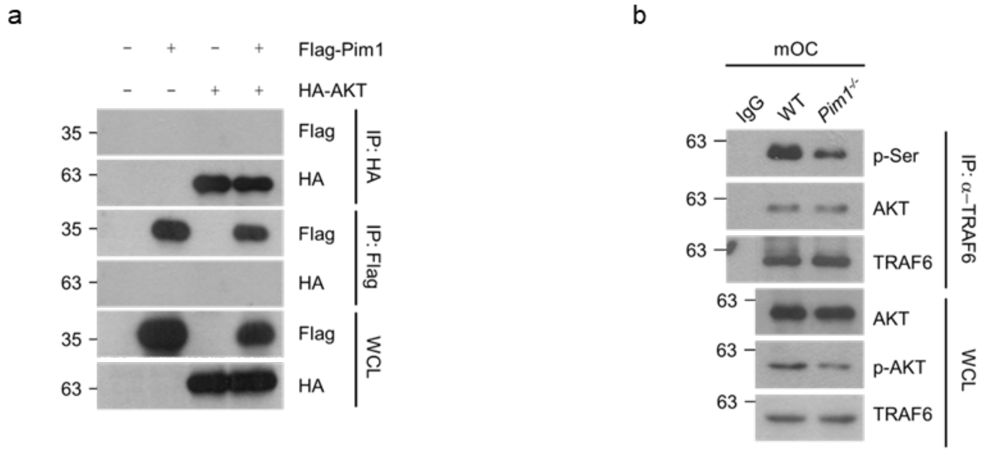

Supplementary Fig. 5. Pim1 does not directly interact with AKT and does not affect the direct interaction between TRAF6 and AKT.

(a) HEK293T cells were transfected with the indicated combinations of expression plasmids, followed by co-immunoprecipitation with an anti-HA and an anti-Flag antibody. The proteins in the immunoprecipitates were detected by immunoblotting with the indicated antibodies. The expression levels of the transfected plasmids were verified by immunoblot analysis of the whole-cell lysates. Representative immunoblots are shown. (b) Cell lysates from mature WT and Pim1$^{-/-}$ osteoclasts were subjected to immunoprecipitation with an anti-TRAF6 antibody. The proteins in the total cell lysates were detected by immunoblotting with the indicated antibodies.

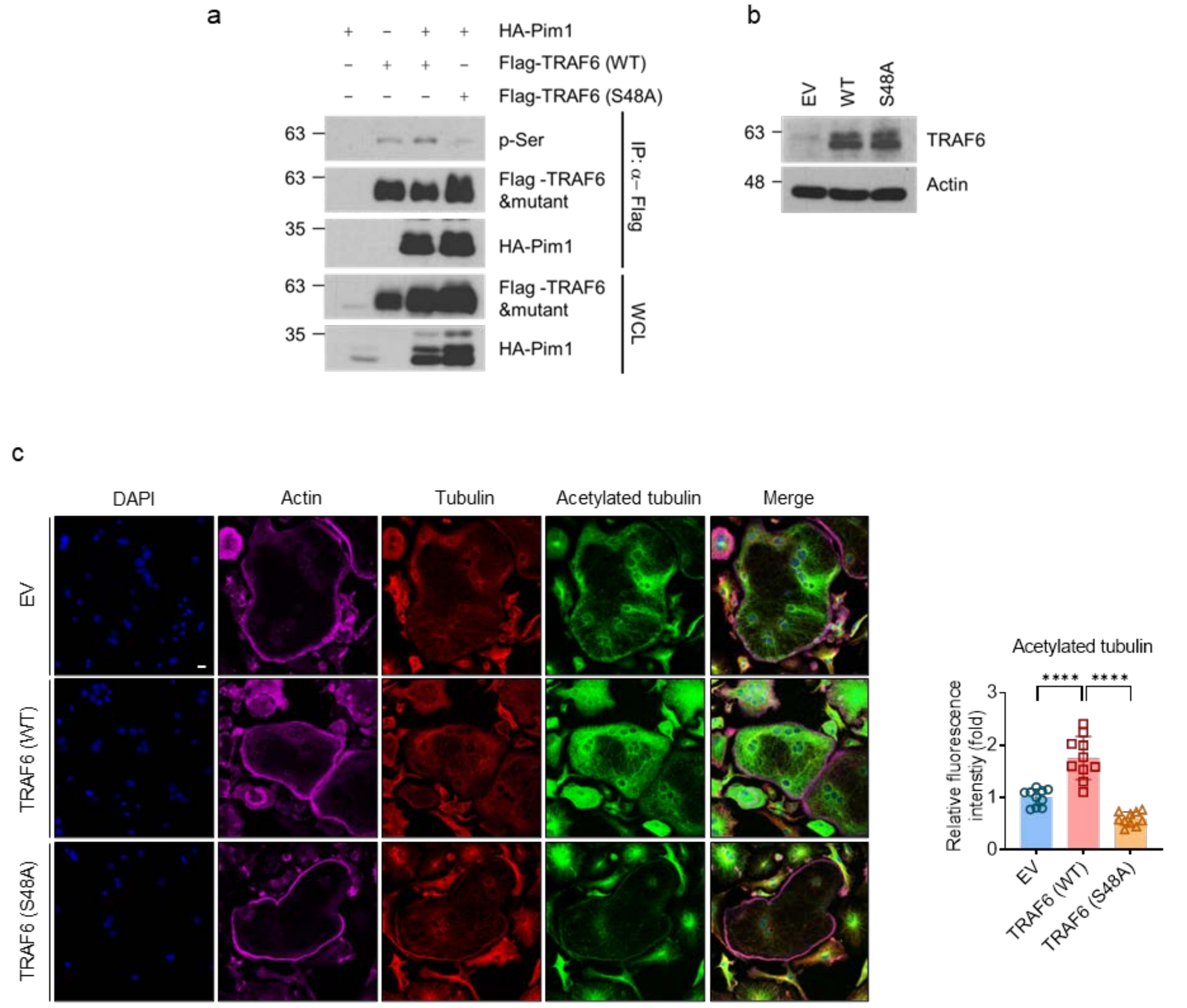

Supplementary Fig. 6. Pim1 phosphorylates TRAF6 at serine 48, subsequently mediating microtubule acetylation in osteoclasts.

(a) HEK293T cells were transfected with the indicated combinations of expression plasmids, followed by co-immunoprecipitation with an anti-Flag antibody. The proteins in the immunoprecipitates were detected by immunoblotting with the indicated antibodies. The expression levels of the transfected plasmids were verified by immunoblot analysis of the whole-cell lysates. Representative immunoblots are shown. (b,c) BMMs were transduced with empty vector (EV), TRAF6 (WT) or TRAF6 (S8A) and incubated with M-CSF (30 ng/ml) and RANKL (100 ng/ml) prior to culture on 96 well polystyrene microplate. (b) Immunoblot analysis of TRAF6 levels in the cell lysate. (c) The cells on 96 well polystyrene microplate

were incubated with antibodies specific for tubulin or acetylated tubulin followed by Alexa Fluor 594 (bright red)- and Alexa Fluor 488 (green)-labeled secondary antibodies, respectively. F-actin was also stained with Alexa Fluor 647-phalloidin (purple). Representative images are shown. Scale bars, 20 μm. The data are presented as mean ± SD. Statistical analysis was conducted with one-way ANOVA with Turkey's multiple comparison test. ns, not significant. ****$p<0.0001$.

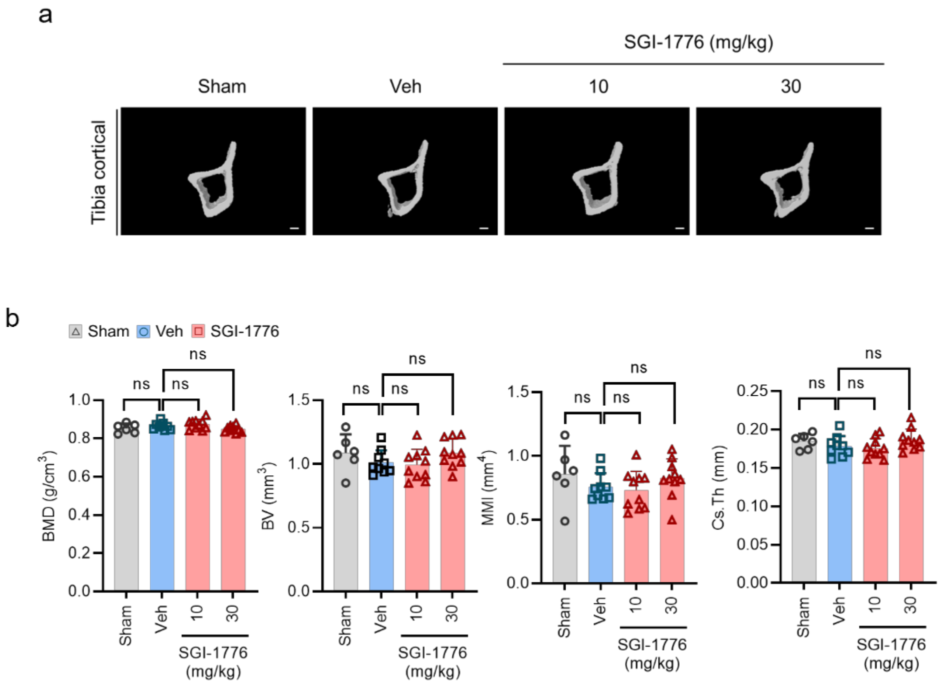

Supplementary Fig. 7. SGI-1776 has no effect on the ability of the tibially injected RM1 prostate-cancer cells to lyse tibial cortical bone in vivo.

Seven-week-old male mice were injected with either PBS, RM1 cells alone, or RM1 cells combined with either 10 mg/kg or 30 mg/kg body weight SGI-1776 (**a**) Representative µCT images. (**b**) Quantitative µCT analysis of the cortical-bone variables in the tibiae. BMD=cortical bone-mineral density; BV=cortical bone volume; Cs.Th=cortical crossectional thickness; MMI=mean polar moment of inertia. The data indicated as mean ± SD. Two-tailed unpaired Student's t-test was conducted for statistical analysis. The data are presented as mean ± SD. Statistical differences were determined by using one-way ANOVA with Tukey's multiple comparisons test. ns, not significant.

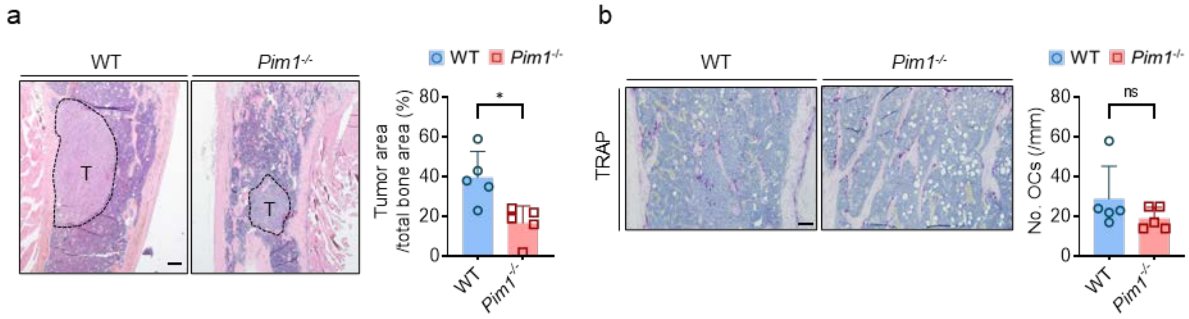

Supplementary Fig. 8. Pim1 deficient mice exhibit reduced tumor-associated osteolysis.

Seven-week-old WT (n=5) and *Pim1*$^{-/-}$ (n=7) male mice were used. Seven-week-old male mice were injected with RM1 cell. **(a, b)** The tibiae were collected on day 9, sectioned, and subjected to (a) hematoxylin and eosin staining or (b) TRAP staining with histomorphometry to determine the tumor area relative to total bone area and the number of osteoclasts per bone perimeter (No.OCs). The data are presented as mean ± SD. Statistical analysis was conducted with two-tailed unpaired Student's t-test. ns, not significant. *$p<0.05$.
